# Local therapies for breast cancer

**DOI:** 10.1007/s12254-017-0336-2

**Published:** 2017-06-07

**Authors:** Ruth Exner

**Affiliations:** Department of Surgery, Medical University of Vienna, Waehringer Guertel 18–20, 1090 Vienna, Austria

**Keywords:** Neoadjuvant therapy, Breast cancer surgery, Reconstruction, Sentinel lymph node

## Abstract

During the San Antonio Breast Cancer Symposium in December 2016, the main topics were systemic treatment of breast cancer and molecular research. But several studies were also presented concerning local therapy: Surgical issues on evaluating resection margins, management of ductal carcinoma in situ (DCIS), surgical challenges after neoadjuvant therapy related to assessment of response or treatment of axillary lymph nodes, and studies about outcome after breast reconstruction and radiation therapy were discussed. In this short review, oral presentations of these topics are summarized.

## Margins

In breast cancer patients undergoing breast-conserving surgery, clear margins are necessary to avoid local recurrence. In recent years, pathology and breast imaging have improved substantially and systemic therapy has also had a major impact on local control.

Before 2014, 50% of additional surgeries for no tumor on ink were performed to ensure wider margins as required by previous standard [1]. Therefore the SSO-ASTRO Consensus Guidelines was published for resection considering there is no need for wider margins other than “no tumor on ink” in invasive cancer [2–4]. Monica Morrow presented data from the Memorial Sloan Kettering Cancer Center (MSKCC) in New York comparing re-excision rate before and after the publication of the guideline showing a significant reduction from 21 to 15% [5]. In addition, preliminary data pointed out the trend in surgical treatment of breast cancer in the years 2013–2015 of a 16% decrease of additional surgery after lumpectomy due to a decrease in the proportion of patients with negative margins having re-excision.

The overall increase in breast-conserving surgery of 13% reflected a decline in both uni- and bilateral mastectomy.

## Postmastectomy radiation therapy and reconstruction

**Fig. 1:**
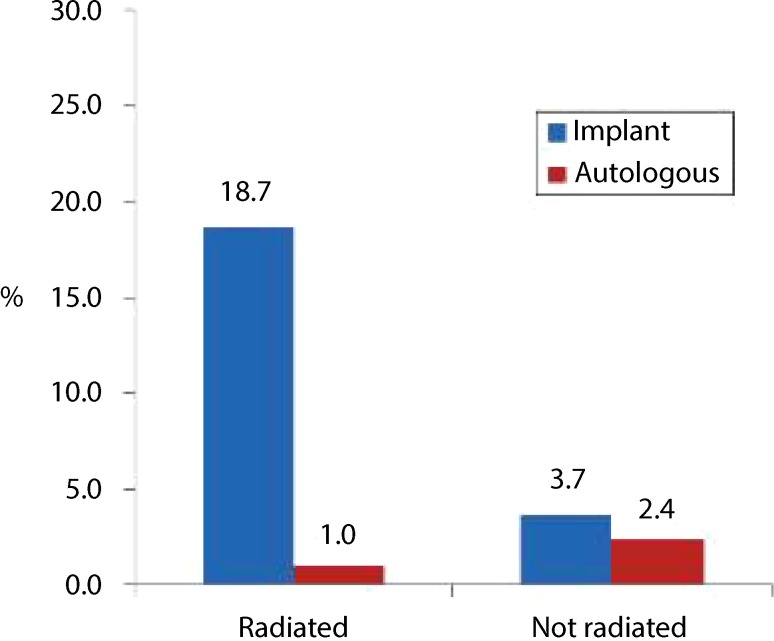
Reconstruction failure. By two years, reconstruction failure occured in 11.4% of radiated patients and 3.4% of non-radiated patients

Radiation therapy can compromise the viability and cosmesis of breast reconstruction concerning skin changes, vascular compromise, fibrosis, and may therefore require repeated surgery for correction. Resma Jagsi, a radiation oncologist from the University of Michigan, Ann Arbor, reported from the prospective multicenter cohort study (MROC study) about the impact of radiation therapy on complications and patient-reported outcomes after breast reconstruction. In this study, 552 radiated and 1461 nonradiated patients after implant or autologous reconstruction were included from 2012–2015. Study end points were complications, reconstruction failures, and patient-reported outcomes after 2 years. Complications like wound infection or hematoma were significantly more common in radiated patients (33.4 vs. 23.5%). Bilateral reconstruction and higher body mass index were predictive of developing a complication, and autologous reconstruction was associated with a lower risk of developing a complication in the radiated patients compared to implant-based reconstruction (*p* = 0.007). Reconstructive failure occurred in 11.4% of the radiated vs. 3.4% of the nonradiated patients. Patient-reported outcomes were better without radiation therapy and superior to autologous reconstruction. This is the largest prospective multicenter study of outcome of breast reconstruction to date and concludes that autologous reconstruction is better for women who are candidates for postmastectomy radiation therapy because of superior patient satisfaction and lower complication rates. Nevertheless, not all women are candidates for autologous reconstruction and also timing may play an important role (Fig. 1).

## Radioactive seed localization

Radioactive seed localization for nonpalpable breast lesions is a promising new technique in breast cancer surgery [6]. Guided by ultrasound, a small titanium seed containing typically 1–10 MBq of radioactive iodine-125 is placed in the center of the nonpalpable breast lesion on the day before surgery. During the operation, the seed is located with a hand-held gamma probe. An interesting study presented by Linnea Langhans from Copenhagen compared radioactive localization versus wire-guided localization of nonpalpable invasive and in situ breast cancer in a Danish randomized multicenter controlled trial: There was no difference in procedure duration, pain, complications, or sentinel node identification rates. Moreover, the new technique was more convenient for patients and surgeons and highly preferred for localizing axillary nodes.

## DCIS (ductal carcinoma in situ) score

The National Comprehensive Cancer Network (NCCN) Guidelines 2016 [7] recommend that if patient and physician perceive individual risk as being low, some ductal carcinoma in situ (DCIS) patients may be treated with excision only, without radiotherapy. A group from Nashville, Tennessee, assessed the relationship between ipsilateral breast event risk and biological risk signature. This signature was developed from three cohorts in the US and Sweden integrating immunohistochemistry biomarkers and clinicopathological features of low and elevated risk DCIS patients to a continuous score. A total of 455 patients were included in the study; follow-up was 10.4 years. Ipsilateral local recurrence in the low-risk group treated with surgery only was 10% and 5% in the group surgery with radiation therapy, while in the elevated risk group it was 30% vs. 10%. These results were similar to studies conducted with the DCIS recurrence score. Two ongoing studies in Australia and Sweden will provide more information about the value of radiotherapy in DCIS in the near future.

## Surgical challenges after neoadjuvant chemotherapy

Tari A. King from Harvard Medical School talked about the consequences of neoadjuvant chemotherapy (NCT) on breast cancer surgery, allowing breast conservation and reduced need for axillary node dissection by downstaging of the disease. On the basis of an interdisciplinary approach, accurate imaging tools for quantifying response are needed.

Comparing residual imaging tumor size to pathologic tumor size, magnetic resonance imaging sensitivity was 86–92%, specificity 60–86% with an accuracy of 90% [8].

Unfortunately increasing rates of pathologic complete regression after NCT have not translated into increasing rates of breast-conserving treatment yet; the causes may be multifactorial:

- Evaluating the extend of residual disease remains a problem
- Resection and detailed pathology review is often the only way to determine suitability for breast-conserving treatment
- Persistent finding of scattered, viable tumor in resection specimen should prompt consideration of re-excision
- Need a standard method for monitoring response, consider difference in response by subtype

## Sentinel and axillary dissection after NCT

Axillary management after NCT remains a challenge. In about 40% of clinically node-positive (cN1) patients, downstaging occurs, which gives the potential to consider sentinel node biopsy only and avoid complete axillary node dissection. In Her2-positive patients, the rate of complete pathological response in the lymph nodes rises to 74%.

The Alliance clinical trial showed that after NCT sentinel node biopsy of primarily positive axillary lymph nodes is feasible: the false-negative rate was 10.7% and detection rate was 84.8% [9].

Suggestions to avoid false-negative rates of sentinel node biopsy after NCT are to remove three or more lymph nodes, use dual agent mapping, perform normal exam after NCT (ultrasound, clinical), use clipping of sentinel lymph node before NCT and include immunohistochemistry-detected disease as node positive.

In the multi-institutional GANEA1 trial conducted in 176 patients with T1–2 breast cancer treated with sentinel node biopsy and axillary lymph node dissection after NCT, the false-negative rate was 11.5%, concluding that the sentinel detection rate varies according to pathological response [10].

A prospective study from MSKCC included 288 cN+ patients in the years 2013–2015. Nearly 70% were eligible for sentinel node biopsy after NCT and for 48% axillary dissection was avoided, supporting the role of NCT in reducing the need for axillary dissection among patients presenting with nodal metastases [11].

Performing a targeted axillary dissection by clipping biopsy-proven involved sentinel lymph nodes before NCT was evaluated in a study from the MD Anderson Cancer Center published in the *Journal of Clinical Oncology* in 2016. The clipped node was located using iodine-125 seed localization; the false-negative rate was determined in patients undergoing complete axillary lymph node dissection. The false-negative rate of the clipped node was only 4.2%. In 23% of patients, the clipped node was not the sentinel [12].

Other trials are ongoing concerning post NCT axillary dissection: The ALLIANCE A11202, where patients with a positive sentinel node are randomized between radiotherapy and axillary lymph node dissection, and the NSABP B51/RTOG 1304, where patients with negative nodes are randomized between radiotherapy and no radiotherapy.

The GANEA 2 trial, a prospective multi-institutional French cohort presenting the follow up of T1-3, N0-1 patients operated in the years 2010–2014 after NCT.

**Fig. 2:**
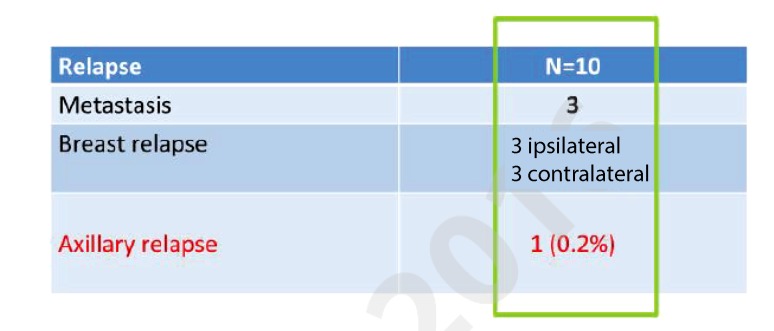
GANEA 2 trail, Group 2 event: 418 patients SLN alone without ALND. Median follow-up = 36 months. *SLN* Sentinel lymph node excision, *ALND* Axillary lymph node dissection

In all, 509 cN0 patients underwent axillary sonography ± fine needle biopsy, the identification rate was 97%, axillary lymph node dissection was not mandatory, breast-conserving rate was 90%, the 3‑year overall survival 97.8%, disease-free survival 94.8%. In all, 418 patients had sentinel node biopsy alone; after a mean follow-up of 36 months, there were 3 patients with distant organ metastases, 3 with ipsilateral and 3 with contralateral breast cancer recurrence, and only 1 axillary relapse (0.2%) (Fig. 2).

The authors claim that before NCT, assessment of axillary status by sonography ± fine needle biopsy is mandatory. In cN+ patients before NCT, sentinel node biopsy alone is not safe outside clinical trials because the false-negative rate is too high, while in N − before NCT sentinel node biopsy seems to be safe, although a study limit was clearly the short follow-up.

The GANEA 3 trial in N + before NCT with tagged initially axillary involved node is already ongoing to avoid unnecessary lymphadenectomy with low false-negative results.

## Conclusions

Surgical management of breast cancer is changing due to effective systemic (neo)adjuvant therapy. There are new techniques to localize nonpalpable lesions, new guidelines regarding resection margins have lowered re-excision rates and targeted axillary dissection after good response to neoadjuvant chemotherapy, has been shown to be safe to avoid complete axillary dissection. After postmastectomy radiation therapy, autologous reconstruction has a better outcome.
